# Drought and Heat Stress Impacts on Phenolic Acids Accumulation in Durum Wheat Cultivars

**DOI:** 10.3390/foods10092142

**Published:** 2021-09-10

**Authors:** Barbara Laddomada, Antonio Blanco, Giovanni Mita, Leone D’Amico, Ravi P. Singh, Karim Ammar, Jose Crossa, Carlos Guzmán

**Affiliations:** 1 Institute of Sciences of Food Production (ISPA), National Research Council (CNR), Via Monteroni, 73100 Lecce, Italy; giovanni.mita@ispa.cnr.it (G.M.); leone.damico@ispa.cnr.it (L.D.); 2 Department of Soil, Plant and Food Sciences, University of Bari ‘Aldo Moro’, Via G. Amendola 165/A, 70126 Bari, Italy; antonio.blanco@uniba.it; 3 International Maize and Wheat Improvement Center (CIMMYT), Global Wheat Program, Apdo Postal 6-641, Ciudad de México 56237, Mexico; R.Singh@cgiar.org (R.P.S.); k.ammar@cgiar.org (K.A.); J.CROSSA@cgiar.org (J.C.); 4 Departamento de Genética, Escuela Técnica Superior de Ingeniería Agronómica y de Montes, Edificio Gregor Mendel, Campus de Rabanales, Universidad de Córdoba, CeiA3, 14071 Córdoba, Spain

**Keywords:** durum grains, phenolic compounds, genetic variability, heritability, climate constraints, yield performance

## Abstract

Droughts and high temperatures are the main abiotic constraints hampering durum wheat production. This study investigated the accumulation of phenolic acids (PAs) in the wholemeal flour of six durum wheat cultivars under drought and heat stress. Phenolic acids were extracted from wholemeals and analysed through HPLC-DAD analysis. Ferulic acid was the most represented PA, varying from 390.1 to 785.6 µg/g dry matter across all cultivars and growth conditions, followed by sinapic acids, *p*-coumaric, vanillic, syringic, and *p*-hydroxybenzoic acids. Among the cultivars, Cirno had the highest PAs content, especially under severe drought conditions. Heat stress enhanced the accumulation of minor individual PAs, whereas severe drought increased ferulic acid and total PAs. Broad-sense heritability was low (0.23) for *p*-coumaric acid but ≥0.69 for all other components. Positive correlations occurred between PA content and grain morphology and between test weight and grain yield. Durum wheat genotypes with good yields and high accumulation of PAs across different growing conditions could be significant for durum wheat resilience and health-promoting value.

## 1. Introduction

Durum wheat (*Triticum turgidum* ssp. *durum* (Desf.) Husnot) is one of the most common cereal crops in Mediterranean climates and the tenth most cultivated species in the world. Despite accounting for only 5% of global wheat production, this wheat species is a key commodity for many areas worldwide, especially for the countries surrounding the Mediterranean basin, North America, the desert area of the southwestern United States, North Mexico, and sub-Saharan Africa [1].

Traditionally, durum-based foods have a large diversification across the producing countries. While pasta is the most popular product worldwide and a symbol of the Italian cousin, couscous is the most common durum-based food in North Africa, and durum wheat breads are traditionally important in Southern Italy, Spain, Turkey, and Mid-East Mediterranean regions. Locally in the Mid-East, durum wheat is also used to make bulgur (made from the cracked parboiled grains) and freekeh (a dish made with green, roasted, and rubbed grains); in Turkey and Cyprus, tarhana is a fermented soup made with durum grains and yogurt or milk. Overall, the above products provide a significant slice of calories and proteins to human diets; additionally, they are an important source of bioactive components contributing to a healthy diet [2,3].

Durum wheat is mainly grown under rainfed conditions, often encountering drought and heat stresses that hamper yield potential and influence the qualitative characteristics of the grains [4,5]. Therefore, it is of great importance to investigate the effects of these environmental constraints that have become more and more frequent due to climate change that is also posing a serious challenge for durum wheat growth [6]. Among the drawbacks of water scarcity in durum cultivation is the reduction of plant height, grain size, transpiration rate, and hormonal imbalance [7,8]. Durum wheat is also very sensitive to elevated temperatures, especially when they occur at grain filling and flowering times [9]. High temperatures can affect its physiological traits and reduce seed germination, grain filling, grain number, photosynthetic capacity, chlorophyll content, and typically induce early leaf senescence [10]. An excess of reactive oxygen species (ROS) production in response to drought and heat stresses can cause damages to proteins, carbohydrates, lipids, and nucleic acids [11]. To counteract the injuries of oxidative stress, the wheat plant has developed a complex defense system based on the production of enzymatic and non-enzymatic antioxidants [12]. Among the latter, phenolic acids are the major subclass of polyphenols participating in the scavenging of ROS in cereals [13]. The antioxidant capacity of these compounds depends on both the number and position of the hydroxyl groups in the benzene ring and on ortho-substitution with the methoxy group [14]. In addition to contributing to biotic and abiotic resistance in plants, in humans, phenolic acids contribute to the prevention of a number of non-communicable diseases due to their anti-inflammatory and anti-carcinogenic properties [3,15].

Typically, phenolic acids are classified as hydroxy derivatives of either cinnamic or benzoic acid, the former including ferulic acid that alone accounts for about 90% of total phenolic acids in the wheat grain [16]. In mature durum kernels, the majority of phenolic acids (up to 75–80%) are insolubly bound to cell wall polymers, while the rest (20–25%) are esterified to sugars and other low molecular mass compounds, and only 0.5–2% are soluble and free [15]. The content and composition of phenolic acids may vary in the wheat germplasm as documented by a large body of literature [17]. The extent of variation for PAs is up to 3.6-fold [18,19], and it is influenced by both the genotype and environmental factors [18,19]. To unravel the genetic base of the trait, investigations were carried out to identify and characterize the enzymatic genes and QTLs involved in phenolic acids pathways [20,21]. To date, only a few reports describe the effect of abiotic and biotic factors on individual phenolic acids accumulation in mature wheat kernels and more studies need to be carried out to support the first evidence [11]. Only a few works have investigated the effects of genotype, environment, and their interaction on the content and composition of phenolic acids in durum wheat grains under drought and heat stress [22,23,24]. Moreover, elite durum wheat cultivars developed from the large gene pool available at the International Maize and Wheat Improvement Center (CIMMYT) grown under abiotic stresses were assessed for yield performances and nutritional and qualitative traits but not for phenolic acids accumulation [25,26]. Some of the above cultivars have been used largely in several durum producing areas and are of great interest due to their high yield performances, disease resistance, grain quality, and tolerance to drought and heat stresses [25].

The objective of this study was to test the impact of drought and heat stresses on phenolic acids content and composition in the mature grains of a set of durum cultivars representative of CIMMYT durum germplasm. In addition to the variability for individual and total phenolic acids, the study was aimed at assessing the effects of genotypes, growth conditions, and their interactions to assess the possibility of breeding for phenolic compounds.

## 2. Materials and Methods

### 2.1. Plant Materials/Agronomic Trials

Six elite durum wheat varieties, which are some of the most important in 50 years of breeding at CIMMYT, were considered in this study: Mexicali (released in 1975), Yavaros (released in 1979), Altar (released in 1984), Atil (released in 2000), Jupare (released in 2001), and Cirno (released in 2008) (Table S1). The cultivars were sown in 2015–16 and in 2016–2017 crop seasons in Ciudad Obregon, Sonora, in northwestern Mexico. The experimental trials were planted with two replicates in a randomized complete block design under six different growth conditions: (1) drip irrigation in beds; (2) full irrigation in flat beds; (3) full irrigation in beds; (4) mild drought stress; (5) severe drought stress; and (6) severe heat stress. All the trials were planted in November, except for severe heat stress (planted in February). All the plots (6.5 m^2^) had full irrigation (>500 mm) except medium drought stress (300 mm) and severe drought stress (180 mm). Weed, diseases, and insects were all well controlled. In all the trials, N was applied (pre-planting) at a rate of 50 kg of N/ha, and at tillering, 150 additional units of N were applied in all the plots except in severe drought stress (50 N units). At maturity, whole plots were harvested, grain yield was calculated, and 1 kg of grain from each durum line was used for analyzing the quality traits. The meteorology data of the experimental station in Ciudad Obregon was characterized by almost no precipitation during the wheat growing season. Average temperatures were between 12 and 24 °C in March and April, the grain filling time for all treatments, except for plants under heat stress at temperatures between 19 and 28 °C during grain filling in May. Flowering time and physiological maturity in most of the examined cultivars occur at similar times because these genotypes were bred for the same growing area. According to the general growing stages of durum wheat in Ciudad Obregon, drought stress was continuous from stem elongation to grain ripening in moderate and severe drought stress trials. In severe heat stress trial, higher temperatures than in the normal planting time started from shoot elongation and remained in the grain filling stage until ripening.

### 2.2. Grain Traits

The digital image system SeedCount SC5000 (Next Instruments, Australia) was used to calculate 1000 kernel weight (g) and test weight (kg/hL). With the same device other grain morphological traits such as grain length, width, and thickness were obtained. Grain protein content (%) and moisture content were determined by near-infrared spectroscopy (NIR Systems 6500, Foss Denmark) calibrated based on official AACC methods 39–10 and 46–11A, respectively (AACC, 2010). Protein content was adjusted to a 12.5% moisture basis.

### 2.3. Phenolic Acids Analysis

Wholegrain samples were milled at particle size ≤1 mm using a 1093 Cyclotec™ Sample mill (FOSS, Hilleroed, Denmark) to produce wholemeal flour. Milled samples were stored at −20 °C until analysis to protect phenolic acids from degradation. Total phenolic acids (sum of soluble and insoluble fractions) were extracted from wholemeal flour samples according to details previously described [19]. In brief, samples were delipidated twice with hexane, hydrolysed with 2 M NaOH, and acidified with HCl 12 M to pH 2 prior to undergoing ethyl acetate extraction. Extracts were dried under nitrogen flux and dissolved in 200 μL of 80:20 methanol/water and quali-quantitatively analyzed using an Agilent 1100 Series HPLC-DAD system (Agilent Technologies, Santa Clara, CA, USA) equipped with a reversed phase C18 (2) Luna column (Phenomenex, Torrance, CA, USA) (5 µm, 250 × 4.6 mm) at a column temperature of 30 °C. A mobile phase consisting of acetonitrile (A) and 10 mL/L water solution of H_3_PO_4_ (B) was used for the following elution program: isocratic elution, 100% B, 0–30 min; linear gradient from 100% B to 85% B, 30–55 min; linear gradient from 85% B to 50% B, 55–80 min; linear gradient from 50% B to 30% B, 80–82 min; and post time, 10 min before the next injection. The flow rate of the mobile phase was 1 mL/min, and the injection volume was 20 µL. The column temperature was kept at 30 °C. Peaks were identified by comparing their retention times and UV-Vis spectra to those of authentic phenolic standards: *p*-hydroxybenzoic acid, vanillic acid, syringic acid, *p*-coumaric acid, sinapic acid, and ferulic acid (Sigma-Aldrich, Gillingham, U.K.). All phenolic acids were quantified via a ratio to the internal standard (3,5-dichloro-4-hydroxybenzoic acid, Sigma-Aldrich, Gillingham, U.K.) added to every sample and using calibration curves of phenolic acid standards. The wavelengths used for quantification of phenolic acids were 280, 295, and 320 nm. All samples were extracted and analyzed in duplicate, and concentrations of individual phenolic acids were expressed in micrograms per gram of dry matter.

### 2.4. Statistical Analysis

We obtained the Best Linear Unbiased Estimated (BLUE) of the durum wheat lines for each of the response traits using a linear mixed model with the fixed effect containing the growth conditions (established by the field management), year (which reflects the different environmental conditions in the two years of the trial that are independent of the field management), durum wheat lines, and their interactions. We also computed the broad-sense heritability (repeatability) and the genetic correlation matrices for the various traits through a combined analysis of the evaluated durum wheat lines across growth conditions and years.

For BLUEs estimation, the following linear mixed model was used:

(1) $$Y_{ijkl}=\mu +E_{i}+Y_{k}+EY_{ik}+R_{j}\left(EY_{ik}\right)+G_{l}+EG_{il}+YG_{kl\;}+EYG_{ikl\;}+\varepsilon_{ijkl}$$

where *µ* is the general mean, $E_{i}$ is the fixed effects of the growth conditions (*I* = 1,…, s),$\;\;Y_{k}$ represents the fixed effects of the years (*k* = 1.2, ..., *y*), $EY_{ik}$ is the inetraction between growth condition and year, $R_{j}\left(EY_{ik}\right)$ is the effects of the replicates (*j* = 1, 2) within growth condition and year assumed to be identically and independently normally distributed with mean zero and variance *σ^2^_j(i k)_,* the fixed effects of the durum wheat lines are $\;G_{l}$ (*l* = 1,2, ..., *m*), $EG_{il}$ is the line by growth condition interaction, the term $YG_{kl}$ is the line by year interaction, and the triple interaction between the growth conditions, year, and durum wheat lines is denoted by $EYG_{ikl\;}.$ The term $\varepsilon_{ijkl}$ is a random residual associated to the *l*th wheat line in the *j*th replicate within the *i*th growth condition and *k*th year combination and assumed to be identically and independently normally distributed with mean zero and variance *σ^2^_ε_.* The code used for fitting the linear mixed model of Equation (1) was generated using SAS software, Version 9.

The broad-sense heritability was calculated as:

(2) $$H^{2}=\frac{\sigma_{g}^{2}}{\sigma_{g}^{2}+\sigma_{ge}^{2}/nloc+\sigma_{gy}^{2}/nyear+\sigma_{gey}^{2}/nlocyear\;\sigma_{\varepsilon }^{2}/\left(nloc\times nyear\times nrep\right)}$$

where $\sigma_{g}^{2}$, $\sigma_{ge}^{2}$, $\sigma_{gy}^{2},\;\sigma_{gey}^{2},\;$and $\sigma_{\varepsilon }^{2}$ are the genotype, genotype by growth condition interaction, genotype by year interaction, genotype by growth condition and by year interaction, and the error variance components, respectively; nloc, nyear, and nrep are the number of growth conditions; and rg is the number of years and number of replicates, respectively.

The genetic correlation matrices among sites were calculated using equations from Cooper and Delacy [27]: $\rho_{g_{ij}=\frac{\rho_{p_{ij}}}{h_{i}h_{i\prime }}}$ where $\rho_{p_{ij}}$ is the phenotypic correlation among growth condition–year combination *i* and *i*^/^, and $h_{i}$ and $h_{i\prime }\;$are the square roots of the growth condition–year combination *i* and *i*^/^, respectively.

## 3. Results

### 3.1. Grain Yield, Grain Traits, and Phenolic Acid Profile of Durum Wheat Cultivars

**Figure 1:**
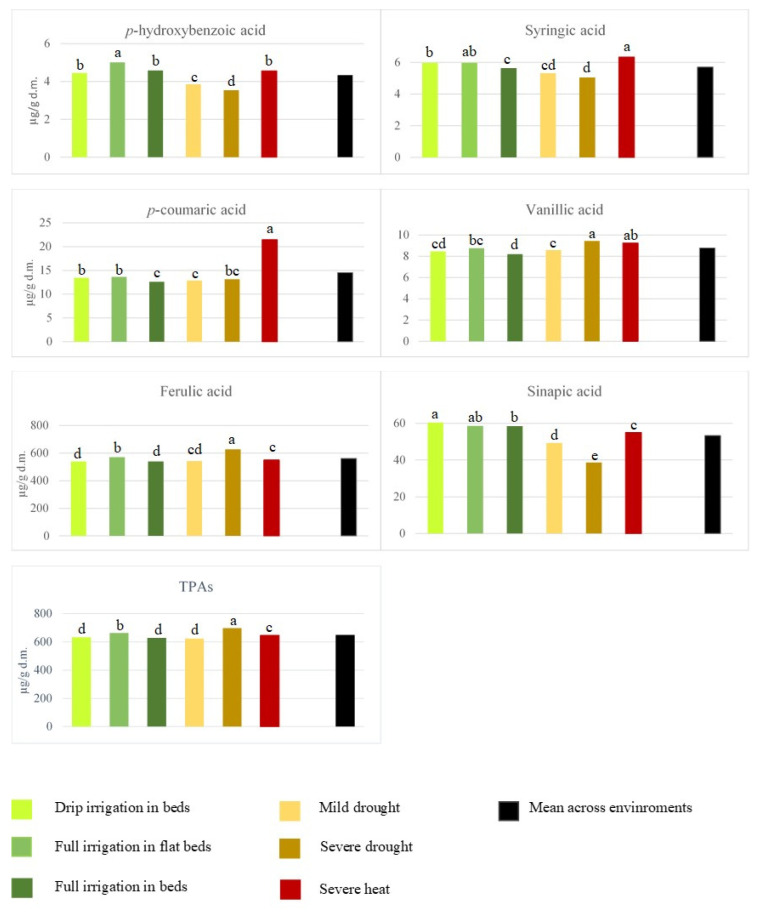
Average values of individual phenolic acids, evaluated in wholemeal flour of six CIMMYT durum cultivars over two years under six different growth conditions. TPAs: total sum of individual phenolic acids. Different letters show significant differences (*p* < 0.05).

Six durum cultivars out the foremost durum varieties developed at CIMMYT were evaluated for grain yield, general grain traits and the content and composition of phenolic acids in wholemeal flour under six growth conditions across two years. The growth conditions varied from optimal to critical due to the effect of moderate to severe drought and severe heat stresses. A summary of the data of grain yield and other grain traits recorded is showed in Table S2. In full irrigation environments, the cultivars produced more than seven tons per hectare, which was reduced to 4 and 3 tons per hectare under severe drought and heat, respectively. Test weight and 1000 kernel weight showed a similar pattern compared to grain yield, while grain protein content was higher in the stressed growth conditions. In overall, the genotype with best performance was Cirno, showing the highest grain yield and size values and the third highest test weight and grain protein content values. A first picture of results of the phenolic acids composition is provided in Table 1 which summarizes the variation of individual phenolic acids of the cultivars grown across all tested conditions and years. Ferulic acid was the most represented phenolic acid with a grand mean of 563.07 µg/g dry matter and a variation range from 390.1 to 785.6 µg/g dry matter across all cultivars and growth conditions. Sinapic acid was the second phenolic acid for abundancy, followed by four minor components (i.e., *p*-coumaric, vanillic, syringic and *p*-hydroxybenzoic acids). Overall, each variety had a typical phenolic acid profile, differing significantly (*p* < 0.05) for almost all individual components (Table 1). Cirno was the cultivar with the highest content of major phenolic acids (i.e., ferulic and sinapic acids) and, so far it had the highest content of total phenolic acids (TPAs). As a second insight into phenolic variation of the cultivars, we looked at the average values of individual phenolic acids as influenced by the six tested growth conditions tested across two years (Table S3). The outcome data revealed an impact of growth conditions that was different on individual phenolic acids (Figure 1, Table S4). While severe heat stress enhanced the accumulation of some minor individual phenolic acids (i.e., *p*-coumaric, syringic and vanillic acids), severe drought had a higher impact on the most abundant phenolic acid, namely ferulic, and consequently on TPAs. By comparing the three main growing conditions used in Mexico for durum wheat (i.e., drip irrigation in beds, full irrigation in flat beds, and full irrigation in beds) with the stress conditions, an increase of individual phenolic acids was found under severe drought (Table S3).

Moreover, the results showed that sinapic acid was not enhanced neither by water scarcity nor by elevated temperatures, but it increased under the drip irrigation in beds, full irrigation in flat beds, and full irrigation in beds conditions (*p* < 0.05). The outcome results also remarked a rise of the two major phenolic acids (i.e., ferulic and sinapic acids), and of the least abundant *p*-hydroxybenzoic acid under the full irrigation in flat beds condition.

### 3.2. Effects of Genotype, Growth Conditions, Year, and Their Interactions

An ANOVA analysis was carried out to determine the effects of the genotype, growth conditions, year, and their interactions on the grain phenolic acid contents. The analysis showed that almost all the factors involved in the experimental trial had a highly significant effect on phenolic acids (*p* < 0.0001) (Table 2). The year and growth conditions were the most impactful sources of variation, followed by genotype and the E × Y, G × E, E × Y and G × E × Y interactions. The Rep (E × Y) interaction was not significant for all the traits. The variance ascribed to the year, due to the effect of the different climatic conditions occurring across the two years of the experimental trials, was particularly high for almost all individual phenolic acids, with the exception of two minor components (i.e., *p*-hydroxybenzoic acid and *p*-coumaric acid). Conversely, genotype variance was consistent for *p*-hydroxybenzoic acid, while the first source of variation for *p*-coumaric acid was the growth conditions (Table 2). Broad-sense heritability, based on the variance component estimates with combined analysis, varied largely among individual phenolic acids, being low (0.23) for *p*-coumaric acid, above 0.69 for all other components, and 0.65 for TPAs (Table 1).

### 3.3. Relationships between Phenolic Acids, Yield Components, and Protein Content

The phenotypic correlation coefficients were calculated among individual phenolic acids, TPAs, and other traits, as shown in Table 3. In particular, we considered the correlations between phenolic acids and grain yield, 1000 kernel weight, test weight, grain length, grain width, and protein content across years and growth conditions. Four of the individual phenolic acids and TPAs were correlated in a positive manner (*p* < 0.05) with grain yield. In addition to this, all individual phenolic acids except vanillic acid showed a positive or neutral correlation with 1000 kernel weight. In the case of test weight, only syringic and sinapic acid showed positive associations with it. TPAs were positively correlated with grain yield and all other kernel morphological traits, except for test weight (Table 3), although, in this case, the correlation was significant but weak (*r* = −0.14). Similarly, the protein content was negatively correlated (*p* < 0.05) with the minor PAs (syringic, vanillic, and *p*-coumaric acids) and positively correlated with the major PAs and TPAs. Moreover, looking at the correlations among individual PAs, *p*-hydroxybenzoic acid had negative correlations with syringic, vanillic, and *p*-coumaric acids and positive correlations with sinapic and ferulic acids and TPAs.

## 4. Discussion

Durum wheat-based foods are major components of the human diet in many areas worldwide, and the content of grain functional compounds, such as phenolic acids that are beneficial to human health, has become an important subject of research. While there is a wide knowledge on the genetic variability of phenolic acids in wheat germplasm collections [17], in view of durum breeding programs, the assessment of the genetic stability of phenolic acids under different growth conditions has been reported only in a few investigations [22,23,24,28]. A recent report released by the Food and Agriculture Organization [29] showed that the increasing frequency and intensity of extreme weather constraints, such as water scarcity and elevated temperatures as a result of climate changes, are having a devastating effect on food security and livelihoods, also posing a challenge to durum wheat cultivation. Our study is the first one affording the effect of drought and heat stress on the phenolic acids profile of a set of durum cultivars developed at CIMMYT over the last 50 years. The examined cultivars have been introduced in many wheat producing areas due to their high yield potential, disease resistance, grain quality, and tolerance to drought and heat stresses, and they have been used by durum breeding programs carried out by different research institutions in several countries [25]. Based on the overall data, we identified six main individual phenolic acids (i.e., ferulic acid, vanillic acid, *p*-coumaric acid, sinapic acid, syringic acid, and *p*-hydroxibenzoic acid), in line with results arisen from durum wheat genetic diversity screen [19] and validating previous findings on the effects of abiotic stress on the accumulation of phenolic acids [23]. The range of variation across the cultivars, years, and growth conditions for phenolic acids was comprised between 444.9 and 902.2 µg/g dry matter. So far, a twofold range of variation was found in this study, which was slightly lower with that observed in the genetic diversity assessment considering a higher number of genotypes [18,19]. This is probably due to the fact that, in our study, we only used modern durum varieties developed by the same breeding program while the cited studies analyzed the variability for phenolic compounds in heterogenous collections with materials of different wheat species and origins.

Compared to previous works which assessed the effects only of elevated temperatures [23,28] or drought [24], this study considered the effects of both drought and heat stress on a set of durum wheat genotypes. Another element of novelty of the present work is that we did not consider the variation for soluble free, soluble bound, and insoluble bound phenolic acids separately, but we considered the variation for all fractions as a whole, as previously shown [19]. This choice was supported by evidence that the amount of total soluble free, soluble bound, and insoluble bound phenolic acids extracted separately is equal to the amount of the three fractions recovered together [15,19]. Moreover, it was shown that the relative proportion of the three fractions in wheat grains (0.5–2% soluble free; 18–22% soluble bound; 77–80% insoluble bound) is common to all wheat genotypes independently of growing conditions and environments [4,19,30]. All phenolic fractions have important biological properties to protect human health. While soluble free phenolic acids are rapidly absorbed by the small intestine and protect against cardiovascular disease and colon cancer due to their antioxidant properties [31], soluble and insoluble bound phenolic acids protect against colon inflammation and cancer [3,32,33]. The occurrence of ester or ether linkages to cell wall polymers or to other low molecular mass components is not a hindrance for phenolic acids to exert their biological activity as they are metabolized locally by the colon microflora [34,35].

Previous works investigating on the effect of water stress on eight durum genotypes found that phenolic acids accumulated differently in the mature grains independently of whether they were resistant, tolerant, or sensitive to stress [24]. Some genotypes did not exhibit any significant change in phenolic acids under water scarcity, while some others had higher concentrations compared to those grown under non-stress conditions [24]. In the current study, the highest TPAs values were found under severe drought conditions, while moderate drought and severe heat stress did not lead in all cases to higher concentrations of PAs compared to the full irrigation growth conditions. This is interesting, particularly in the case of heat stress, because in this growth condition the lowest values of 1000 kernel weight and test weight were registered. One would expect that the smallest and most shrivelled grain would have the highest PAs content because PAs are concentrated in the bran layers.

The absence of this effect under heat stress condition indicates the probable negative effects of heat on the metabolic pathway leading to the production of PAs. A three-way ANOVA was used to evaluate the portion of genetic variation for the individual phenolic acids ascribed to each source of variation. Based on the results, the year and growth conditions had the largest effect on individual and total PAs contents, followed by the genotype and the G × E, G × Y, E × Y and G × E × Y interactions. The results agree with some previous reports and disagree with others. Shamloo et al. [23] found a predominant genotypic effect on the accumulation of total phenolic acids content in mature wheat grains under elevated temperatures. A general increase of phenolic acids was observed, though the different wheat cultivars showed different grades of increase [23]. Other works investigated the impact of terminal heat stress, finding a negative influence on several secondary metabolites of wheat grains, including phenolic acids [28] (Shahid et al., 2017), which agree with our results commented above. Nevertheless, these studies also confirmed a higher effect of the genotype over environmental factors on the observed variation [28]. Conversely, when the effects of several climate parameters were considered, prevalent effects from the environment and the genotype–environment interactions on the total soluble phenolic contents of mature grains were detected [22], similar to what we found. Discrepancies among the different research could be due to the diverse experimental plan of the studies, considering different genetic materials and the highly different climate and agronomical conditions. In addition to this, the heritability values were calculated for all PAs. The heritability estimated for TPAs was 0.65, which confirmed previous reports by studies carried out on durum wheat [19] and higher compared to those observed in bread wheat [30,36]. For ferulic acid, the most abundant PA in the durum grain, heritability was 0.69, indicating that the proportion of the total phenotypic variance that is attributable to the average effects of genes is quite high for these grain compounds, which could make feasible breeding approaches to increase the PA content. Related to this, it was interesting to identify in the correlation analysis that when TPAs increase, the grain length, grain width, and grain thickness also increase and are positively correlated with test weight and grain yield. Indeed, Cirno was the cultivar showing the highest grain yield and TPAs in our trial, indicating that productivity is not at odds with high PA concentration. In previous studies [23,24], it was not clear if PA variation in mature grains caused by heat or drought was an indirect effect of bran to endosperm ratios or grain filling entity or grain size. Our data suggests that, in general, when climate constraints affect grain morphological traits, a concomitant reduction of phenolic accumulation occurs in mature grains.

## 5. Conclusions

In this study, the effect of water scarcity and elevated temperatures on phenolic acids profile of the wholemeal flour of a set of CIMMYT elite durum wheat cultivars was evaluated. The varieties had a typical phenolic acid profile under different growth conditions, differing significantly for almost all individual PA components. Cirno was the cultivar showing the highest content of phenolic acids across years and growth conditions, especially under severe drought conditions. Overall, severe heat stress enhanced the accumulation of minor phenolic acids (i.e., *p*-coumaric, syringic and vanillic acids) and reduced the main ones (i.e., ferulic acid), whereas severe drought had a higher impact on ferulic acid and the total sum of PAs.

The broad-sense heritability varied largely among the individual phenolic acids, being low for *p*-coumaric acid and ≥ 0.69 for all other components, and significant genotype effects were found for all PAs. In addition, positive correlations were found between total PAs and grain morphology parameters, test weight, and grain yield, suggesting that breeding for high-yielding varieties with high PAs concentration is feasible.

## Figures and Tables

**Table 1:** Mean values, minimum/maximum values, coefficient of variation (CV), heritability and least significant difference (LSD) for grain phenolic acids content (µg/g dry matter) in six CIMMYT durum wheat cultivars evaluated across two years and six growth conditions. TPAs: total sum of individual phenolic acids. Numbers with the same letter in each column are not significantly different (*p* < 0.05).

	*p*-Hydroxy-benzoic Acid	Syringic Acid	Vanillic Acid	*p*-Coumaric Acid	Ferulic Acid	Sinapic Acid	TPAs
Altar	4.87 ^b^	5.37 ^d^	8.15 ^c^	14.24 ^bc^	540.96 ^c^	51.58 ^c^	625.18 ^cd^
Atil	4.80 ^b^	5.16 ^d^	8.14 ^c^	12.94 ^d^	563.69 ^b^	55.04 ^b^	649.75 ^b^
Cirno	5.12 ^a^	5.81 ^bc^	8.16 ^c^	13.85 ^c^	611.00 ^a^	59.13 ^a^	703.09 ^a^
Jupare	3.85 ^c^	6.10 ^ab^	9.30 ^b^	14.84 ^b^	530.09 ^c^	54.62 ^b^	618.80 ^d^
Mexicali	3.30 ^d^	6.14 ^a^	9.83 ^a^	14.09 ^b^	568.34 ^b^	54.74 ^b^	657.29 ^b^
Yavaros	3.99 ^c^	5.73 ^c^	9.06 ^b^	16.38 ^a^	559.15 ^b^	45.21 ^d^	639.52 ^bc^
Grand Mean	4.34	5.72	8.79	14.52	563.07	53.2	649.87
Range	1.9–6.8	2.4–8.5	5.4–12.8	7.4–38.3	390.1–785.6	29.4–92.3	444.9–902.2
CV	6.9	9.87	7.48	8.3	4.92	5.16	4.86
Heritability	0.95	0.75	0.89	0.23	0.69	0.85	0.65
LSD	0.17	0.33	0.38	0.74	17.1	2.21	19.51

**Table 2:** Analysis of variance of individual phenolic acids in six CIMMYT durum wheat cultivars evaluated across two years and six growth conditions. Mean squares values are shown.

Source	DF	*p*-Hydroxy Benzoic Acid	Syringic Acid	Vanillic Acid	*p*-Coumaric Acid	Ferulic Acid	Sinapic Acid	TPAs ^1^
Year (Y)	1	0.61 *	59.82 ***	83.31 ***	0.21 ***	220,684.87 ***	6149.96 ***	320,586.19 ***
Growth conditions (E)	5	7.08 ***	5.51 ***	5.26 ***	276.34 ***	27,885.92 ***	1650.95 ***	18,322.21 ***
E x Y	5	4.01 ***	5.11 ***	3.10 ***	32.38 ***	19,815.92 ***	540.06 ***	27,821.01 ***
Rep (E x Y)	12	0.08 ns	0.26 ns	0.44 ns	0.45 ns	214.82 ns	9.10 ns	284.14 ns
Genotype (G)	5	12.07 ***	3.73 ***	12.13 ***	32.43 ***	20507.17 ***	545.30 ***	24,087.22 ***
G × Y	5	0.28 ***	0.214 ***	1.380 *	4.117 *	9046.34 ***	109.01 ***	11,060.94 ***
G × E	25	0.41 *	1.28 ***	1.46 ***	45.12 ***	6831.27 ***	82.69 ***	9043.75 ***
G × E × Y	25	0.95 ***	0.74 **	1.26 ***	9.16 ***	5768.96 ***	95.32 ***	7721.88 ***
Error	60	0.09	0.33 ***	0.43 ***	1.65 ***	876.77 ***	14.71 ***	1141.52 ***

^1^: sum of individual phenolic acids. *: *p* < 0.05, **: *p* < 0.01, ***: *p* < 0.001, ns = not significant, DF = degree of freedom.

**Table 3:** Correlation coefficients (*r*) among individual phenolic acids (PAs) contents and other phenotypic traits including yield components protein content and kernel morphology and across year and growth conditions. TPAs: total sum of individual phenolic acids.

	Grain Yield	1000 K. Weight	Test Weight	Protein Content	Grain Length	Grain Width	Grain Thickness	*p*-Hydroxy Benzoic Acid	Syringic Acid	Vanillic Acid	*p*-Coumaric Acid	Ferulic Acid	Sinapic Acid	TPAs
Grain yield	1													
1000 K. weight	0.78	1												
Test weight	−0.16	−0.60	1											
Protein content	NS	−0.13	0.43	1										
Grain length	−0.39	−0.62	0.88	0.69	1									
Grain width	NS	−0.32	0.64	−0.38	0.24	1								
Grain thickness	−0.60	−0.91	0.64	−0.17	0.48	0.66	1							
*p*-Hydroxybenzoic	0.55	NS	0.24	0.46	NS	NS	NS	1						
Syringic	0.17	0.39	NS	−0.62	−0.25	0.47	NS	−0.64	1					
Vanillic	−0.42	NS	−0.18	−0.51	NS	0.12	NS	−0.98	0.78	1				
*p*-Coumaric	−0.49	−0.36	NS	−0.84	−0.23	0.53	0.59	−0.64	0.54	0.65	1			
Ferulic	0.32	−0.26	0.87	0.47	0.64	0.59	0.35	0.62	−0.15	−0.52	−0.34	1		
Sinapic	0.77	0.65	0.13	0.55	0.13	−0.13	−0.66	0.43	NS	−0.33	−0.79	0.46	1	
TPAs	0.41	−0.14	0.83	0.49	0.61	0.56	0.24	0.61	NS	−0.50	−0.40	0.99	0.56	1

All correlations are significant (*p* < 0.05) except for NS: not significant.

## Data Availability

The data is contained within the article.

## Supplementary Materials

The following are available online at https://www.mdpi.com/article/10.3390/foods10092142/s1; Table S1. List and pedigree of the CIMMYT durum wheat cultivars; Table S2. Mean and range of values for different traits in six CIMMYT durum wheat cultivars evaluated across two years and six environments; Table S3. Individual phenolic acids (µg/g dry matter) in six CIMMYT durum wheat cultivars evaluated across two years and six growing conditions; Table S4. Means of individual and total phenolic acids in six CIMMYT durum wheat cultivars evaluated across two years and six growing conditions. TPAs: total sum of individual phenolic acids. Different letters between columns indicate significant differences (*p* < 0.05).
