# Supplementary material for: Drought and Heat Stress Impacts on Phenolic Acids Accumulation in Durum Wheat Cultivars
Source: Foods. 2021 Sep 10;10(9):2142. doi: 10.3390/foods10092142 (PMC8468590; doi:10.3390/foods10092142)
Supplement: Supplementary file 1 [file foods-10-02142-s001.zip › foods-1341612-supplementary.pdf]

Table S1. List and pedigree of the CIMMYT durum wheat cultivars.

Cultivar	Year of release	Pedigree	Country of origin
**Altar**	1984	RUFF/FLAMINGO,MEX//MEXICALI-75/3/SHEARWATER	Mexico
**Atil**	2000	SOOTY-9/RASCON-37	Mexico
**Cirno**	2008	SOOTY-9/RASCON-37//CAMAYO	Mexico
**Jupare**	2001	STINKPOT//ALTAR-84/ALONDRA	Mexico
**Mexicali**	1975	GERARDO-VZ-469/3/JORI(SIB)//ND-61-130/LEEDS	Mexico
**Yavaros**	1979	JORI-69(SIB)/(SIB)ANHINGA//FLAMINGO	Mexico

Table S2. Mean and range of values for different traits in six CIMMYT durum wheat cultivars evaluated across two years and six environments.

	Grain yield (t/ha)		1000 kernel weight (g)		Test weight (kg/hL)		Grain length (mm)		Grain width (mm)		Grain thickness (mm)		GPC[4] (%)	
	Mean	Range	Mean	Range	Mean	Range	Mean	Range	Mean	Range	Mean	Range	Mean	Range
Altar[1]	6.1	2.1-10.2	44.7	31.1-56.5	82.7	78.8-85.2	7.3	6.9-7.5	3.2	2.7-3.5	3.1	2.7-3.4	13.3	10.9-17.5
Atil	5.8	1.6-10.5	46.1	33.5.55.9	81.1	75.6-84.1	7.5	7.3-7.8	3.2	2.9-3.4	3.2	2.8-3.4	14.4	10.2-18.9
Cirno	6.7	2.2-11	48.4	34.5-59.1	81.7	76.3-84.5	7.4	7.2-7.9	3.3	2.9-3.6	3.2	2.9-3.5	13.9	9.7-20.6
Jupare	6.4	2.4-9.7	43.6	33.2-50.2	82.9	79.1-85.0	7.2	6.9-7.4	3.3	2.9-3.4	3.2	2.8-3.4	13.2	10.7-17.7
Mexicali	5.6	1.8-9.3	47.7	35.6-58.2	81.2	77.5-84.1	7.5	7.3-7.9	3.3	2.9-3.6	3.2	2.9-3.4	13.9	10.7-18.9
Yavaros	5.4	1.2-8.7	46.8	34.7-56.5	80.1	75.7-82.4	7.4	7.2-7.8	3.3	2.9-3.5	3.2	2.9-3.4	13.2	10.7-17.8
Cycle 15-16[2]	5.56	1.2-10.4	82.0	31.1-59.1	48.7	75.7-85.2	7.4	6.9-7.9	3.3	2.7-3.6	3.2	2.7-3.4	13.8	9.7-20.6
Cycle 16-17	6.47	2.4-11.0	81.3	33.5-51.4	43.7	75.6-84.4	7.3	7.0-7.6	3.1	2.8-3.4	3.1	2.8-3.5	13.5	11.8-17.2
Drip irrigation in beds[3]	7.9	5.5-10.4	49.2	41.5-58.2	82.5	81.1-84.5	7.4	7.2-7.6	3.4	3.2-3.6	3.3	3.2-3.5	13.1	12.2-14.3
Full irrigation in flat beds	7.6	6.1-9.0	47.1	43.0-51.5	82.9	79.9-85.0	7.3	7.0-7.5	3.3	3.1-3.4	3.2	3.1-3.3	12.1	10.2-14.6
Full irrigation in beds	8.0	5.6-11.0	50.6	44.9-59.1	83.5	81.5-85.2	7.4	7.2-7.7	3.4	3.1-3.6	3.3	3.2-3.4	12.1	9.7-13.8
Mild drought	5.3	3.3-7.4	48.8	39.0-58.7	82.0	79.4-83.9	7.5	7.2-7.9	3.3	3.0-3.5	3.2	3.0-3.4	14.2	12.6-16.2
Severe drought	4.1	1.6-5.1	44.2	31.1-46.0	78.5	75.6-80.6	7.3	6.9-7.5	3.0	2.7-3.2	2.9	2.7-3.2	16.9	13.9-20.6
Severe heat	3.0	1.2-5.7	37.3	37.0-55.1	80.5	75.9-82.9	7.3	7.0-7.7	3.2	3.0-3.5	3.1	2.9-3.4	13.6	12.4-16.2
Heritability	0.83		0.77		0.95		0.95		0.65		0.63		0.85	

[1]Means values are averaging years and environments; [2]Mean values are averaging genotypes and environments; [3]Mean values are averaging genotypes and years; [4]Grain protein content

Table S3. Individual phenolic acids (µg/g dry matter) in six CIMMYT durum wheat cultivars evaluated across two years and six growing conditions.

Individual phenolic acids	Cultivar	Year	Drip irrigation in beds	Full irrigation in flat beds	Full irrigation in beds	Mild drought	Severe drought	Severe heat	Mean of environments
p-Hydroxybenzoic acid	ALTAR	2016	5.29	7.29	4.46	4.12	3.31	4.79	4.88
		2017	4.43	4.26	5.51	4.44	4.81	5.87	4.88
		Mean years	*4.86*	*5.77*	*4.98*	*4.28*	*4.06*	*5.33*	*4.88*
	ATIL	2016	4.35	6.27	4.74	4.23	3.55	4.98	4.69
		2017	5.18	4.86	5.40	4.42	4.20	5.43	4.91
		Mean years	*4.76*	*5.56*	*5.07*	*4.32*	*3.87*	*5.20*	*4.80*
	CIRNO	2016	5.10	5.10	5.09	3.26	5.37	5.04	4.83
		2017	5.11	5.69	5.01	6.69	4.33	5.68	5.42
		Mean years	*5.10*	*5.39*	*5.05*	*4.97*	*4.85*	*5.36*	*5.12*
	JUPARE	2016	3.85	4.80	3.74	3.52	2.75	4.17	3.80
		2017	4.25	4.28	3.33	3.84	3.04	4.74	3.91
		Mean years	*4.05*	*4.54*	*3.53*	*3.68*	*2.89*	*4.45*	*3.86*
	MEXICALI	2016	3.84	4.74	3.73	3.08	2.07	3.07	3.42
		2017	3.06	3.16	4.56	2.70	2.57	3.39	3.24
		Mean years	*3.45*	*3.95*	*4.14*	*2.89*	*2.32*	*3.23*	*3.33*
	YAVAROS	2016	5.21	5.61	3.96	2.76	2.49	3.52	3.92
		2017	3.67	3.99	5.47	3.18	3.93	4.12	4.06
		Mean years	*4.44*	*4.80*	*4.71*	*2.97*	*3.21*	*3.82*	*3.99*
	Mean cultivars		***4.44***	***5.00***	***4.58***	***3.85***	***3.53***	***4.57***	***4.33***
Syringic acid	ALTAR	2016	4.94	6.00	3.80	3.96	3.37	6.51	4.76
		2017	6.39	6.26	6.85	4.56	5.57	6.06	5.95
		Mean years	*5.66*	*6.13*	*5.32*	*4.26*	*4.47*	*6.29*	*5.35*
	ATIL	2016	4.47	4.33	3.57	4.47	3.67	5.55	4.34
		2017	6.43	6.58	6.10	5.31	5.21	6.27	5.98
		Mean years	*5.45*	*5.45*	*4.84*	*4.89*	*4.44*	*5.91*	*5.16*
	CIRNO	2016	4.35	4.77	4.52	5.71	4.92	6.30	5.10
		2017	6.15	6.88	6.25	7.47	5.69	6.70	6.52
		Mean years	*5.25*	*5.82*	*5.38*	*6.59*	*5.30*	*6.50*	*5.81*

	JUPARE	2016	5.43	4.96	4.27	6.14	4.53	7.62	5.49
		2017	7.91	7.55	6.28	6.41	5.29	6.86	6.71
		Mean years	*6.67*	*6.25*	*5.27*	*6.27*	*4.91*	*7.24*	*6.10*
	MEXICALI	2016	6.22	6.16	5.20	5.42	4.85	5.76	5.60
		2017	7.26	6.00	8.29	5.59	6.99	5.97	6.68
		Mean years	*6.74*	*6.08*	*6.74*	*5.50*	*5.92*	*5.86*	*6.14*
	YAVAROS	2016	5.65	6.25	4.33	3.88	4.16	6.29	5.09
		2017	6.44	5.97	8.32	4.91	6.28	6.23	6.36
		Mean years	*6.04*	*6.11*	*6.33*	*4.40*	*5.22*	*6.26*	*5.73*
	Mean cultivars		***5.97***	***5.97***	***5.65***	***5.32***	***5.04***	***6.34***	***5.72***
Vanillic acid									
	ALTAR	2016	7.09	8.88	6.42	6.53	7.79	8.29	7.50
		2017	8.21	8.14	8.65	8.49	9.53	9.78	8.80
		Mean years	*7.65*	*8.51*	*7.54*	*7.51*	*8.66*	*9.04*	*8.15*
	ATIL	2016	6.63	6.30	6.00	7.13	8.38	7.50	6.99
		2017	9.42	8.76	8.83	9.14	10.22	9.28	9.28
		Mean years	*8.02*	*7.53*	*7.41*	*8.14*	*9.30*	*8.39*	*8.13*
	CIRNO	2016	6.78	7.42	6.25	7.72	9.84	7.92	7.65
		2017	8.02	8.73	7.93	9.44	9.28	8.65	8.67
		Mean years	*7.40*	*8.08*	*7.09*	*8.58*	*9.56*	*8.28*	*8.16*
	JUPARE	2016	8.57	8.61	7.21	9.04	9.11	8.27	8.47
		2017	10.60	10.53	8.88	10.95	9.33	10.49	10.13
		Mean years	*9.58*	*9.57*	*8.05*	*9.99*	*9.22*	*9.38*	*9.30*
	MEXICALI	2016	9.08	9.07	7.85	9.32	8.82	9.77	8.99
		2017	10.30	9.41	11.11	9.81	12.46	11.02	10.68
		Mean years	*9.69*	*9.24*	*9.48*	*9.56*	*10.64*	*10.39*	*9.84*
	YAVAROS	2016	8.15	9.89	6.87	7.51	8.24	9.83	8.41
		2017	8.74	9.17	12.34	7.77	10.16	10.05	9.70
		Mean years	*8.44*	*9.53*	*9.61*	*7.64*	*9.20*	*9.94*	*9.06*
	Mean cultivars		***8.47***	***8.74***	***8.19***	***8.57***	***9.43***	***9.24***	***8.77***
p-Coumaric acid									
	ALTAR	2016	15.22	16.11	12.29	13.33	11.69	17.28	14.32
		2017	12.20	10.64	13.34	12.73	14.20	21.84	14.16
		Mean years	*13.71*	*13.37*	*12.81*	*13.03*	*12.94*	*19.56*	*14.24*

	ATIL	2016	13.27	13.86	9.83	12.16	13.03	15.25	12.90
		2017	13.25	10.61	12.85	13.27	11.42	16.53	12.99
		Mean years	*13.26*	*12.23*	*11.34*	*12.72*	*12.23*	*15.89*	*12.94*
	CIRNO	2016	12.89	14.52	13.78	11.03	15.25	13.85	13.55
		2017	12.65	13.94	13.69	14.51	11.86	18.45	14.18
		Mean years	*12.77*	*14.23*	*13.73*	*12.77*	*13.55*	*16.15*	*13.87*
	JUPARE	2016	8.64	11.95	8.57	13.35	11.23	31.49	14.20
		2017	12.25	11.12	10.57	11.75	9.00	38.13	15.47
		Mean years	*10.44*	*11.53*	*9.57*	*12.55*	*10.12*	*34.81*	*14.84*
	MEXICALI	2016	15.27	17.84	12.24	15.59	13.31	16.56	15.13
		2017	13.11	10.60	14.84	12.79	15.69	21.04	14.68
		Mean years	*14.19*	*14.22*	*13.54*	*14.19*	*14.50*	*18.80*	*14.90*
	YAVAROS	2016	19.51	18.41	12.05	12.79	13.69	24.97	16.90
		2017	13.43	13.73	17.67	11.42	17.24	21.68	15.86
		Mean years	*16.47*	*16.07*	*14.86*	*12.10*	*15.46*	*23.33*	*16.38*
	Mean cultivars		***13.47***	***13.61***	***12.64***	***12.89***	***13.13***	***21.42***	***14.53***
Ferulic acid									
	ALTAR	2016	452.69	581.27	459.34	418.36	581.57	509.75	500.49
		2017	600.62	534.32	568.00	560.93	628.38	596.34	581.43
		Mean years	*526.65*	*557.79*	*513.67*	*489.64*	*604.97*	*553.04*	*540.96*
	ATIL	2016	435.50	539.37	475.21	467.70	553.59	537.98	501.56
		2017	674.88	580.45	627.46	658.30	647.23	566.51	625.80
		Mean years	*555.19*	*559.91*	*551.33*	*563.00*	*600.41*	*552.24*	*563.68*
	CIRNO	2016	516.44	597.41	453.82	514.03	786.80	521.22	564.95
		2017	576.70	674.70	645.54	784.04	669.99	591.38	657.06
		Mean years	*546.57*	*636.05*	*549.68*	*649.04*	*728.39*	*556.30*	*611.00*
	JUPARE	2016	390.36	548.06	398.03	503.19	571.34	453.01	477.33
		2017	588.78	630.22	513.46	597.56	554.67	612.38	582.85
		Mean years	*489.57*	*589.14*	*455.75*	*550.37*	*563.00*	*532.70*	*530.09*
	MEXICALI	2016	531.94	539.06	504.29	558.77	560.86	503.94	533.14
		2017	595.05	519.27	683.68	587.26	699.80	536.17	603.54
		Mean years	*563.49*	*529.16*	*593.98*	*573.02*	*630.33*	*520.05*	*568.34*
	YAVAROS	2016	583.52	603.77	474.18	428.48	631.57	602.21	553.95
		2017	535.77	511.90	665.52	449.98	650.92	572.03	564.35

		Mean years	*559.64*	*557.83*	*569.85*	*439.23*	*641.25*	*587.12*	*559.15*
	Mean cultivars		***540.19***	***571.65***	***539.04***	***544.05***	***628.06***	***550.24***	***562.20***
Sinapic acid	ALTAR	2016	52.60	57.59	44.42	38.62	31.03	59.27	47.26
		2017	65.70	56.10	64.23	51.80	40.96	56.62	55.90
		Mean years	*59.15*	*56.84*	*54.32*	*45.21*	*35.99*	*57.94*	*51.58*
	ATIL	2016	48.41	51.96	42.55	39.85	33.23	60.62	46.10
		2017	83.33	64.69	78.26	63.77	40.38	53.32	63.96
		Mean years	*65.87*	*58.32*	*60.41*	*51.81*	*36.80*	*56.97*	*55.03*
	CIRNO	2016	55.54	59.76	51.18	45.35	44.06	45.66	50.26
		2017	70.43	75.38	74.80	76.97	47.75	62.69	68.00
		Mean years	*62.98*	*67.57*	*62.99*	*61.16*	*45.90*	*54.17*	*59.13*
	JUPARE	2016	42.84	53.66	46.50	48.64	34.55	62.18	48.06
		2017	73.88	69.90	59.26	61.94	39.17	62.95	61.18
		Mean years	*58.36*	*61.78*	*52.88*	*55.29*	*36.86*	*62.56*	*54.62*
	MEXICALI	2016	56.21	53.27	45.56	44.01	32.35	51.27	47.11
		2017	69.01	57.03	88.93	55.98	52.24	51.07	62.37
		Mean years	*62.61*	*55.15*	*67.24*	*49.99*	*42.29*	*51.17*	*54.74*
	YAVAROS	2016	52.34	58.66	37.77	27.73	25.57	46.92	41.50
		2017	54.90	45.12	66.92	37.15	41.77	47.67	48.92
		Mean years	*53.62*	*51.89*	*52.34*	*32.44*	*33.67*	*47.29*	*45.21*
	Mean cultivars		***60.43***	***58.59***	***58.36***	***49.32***	***38.59***	***55.02***	***53.38***
Sum of individual phenolic acids	ALTAR	2016	537.83	677.13	530.72	484.92	638.76	605.90	579.21
		2017	697.54	619.86	666.57	642.94	703.43	696.50	671.14
		Mean years	*617.68*	*648.49*	*598.64*	*563.93*	*671.10*	*651.20*	*625.17*
	ATIL	2016	512.62	622.07	541.90	535.54	615.45	631.88	576.58
		2017	792.50	675.94	738.89	754.20	718.64	657.34	722.92
		Mean years	*652.56*	*649.01*	*640.40*	*644.87*	*667.04*	*644.61*	*649.75*
	CIRNO	2016	601.09	688.99	534.64	587.09	866.23	599.99	646.34
		2017	679.06	785.30	753.21	899.11	748.88	693.54	759.85
		Mean years	*640.07*	*737.14*	*643.93*	*743.10*	*807.56*	*646.76*	*703.09*
	JUPARE	2016	459.68	632.03	468.32	583.87	633.50	566.73	557.35

	2017	697.66	733.59	601.78	692.44	620.49	735.56	680.25
	Mean years	*578.67*	*682.81*	*535.05*	*638.15*	*626.99*	*651.14*	*618.80*
MEXICALI	2016	622.56	630.14	578.86	636.19	622.26	590.36	613.39
	2017	697.78	605.46	811.39	674.12	789.73	628.65	701.19
	Mean years	*660.17*	*617.80*	*695.13*	*655.15*	*705.99*	*609.50*	*657.29*
YAVAROS	2016	674.37	702.58	539.15	483.14	685.71	693.73	629.78
	2017	622.95	589.88	776.24	514.40	730.29	661.78	649.26
	Mean years	*648.66*	*646.23*	*657.69*	*498.77*	*708.00*	*677.75*	*639.52*
Mean cultivars		***632.97***	***663.58***	***628.47***	***624.00***	***697.78***	***646.83***	***648.94***

Table S4. Means of individual and total phenolic acids in six CIMMYT durum wheat cultivars evaluated across two yearsrs and six growing conditions. TPAs: total sum of individual phenolic acids. Different letters between columns indicate significant differences ($p < 0.05$).

	2015/16	2016/17	Drip irrigation in beds	Full irrigation in flat beds	Full irrigation in beds	Moderate drought	Severe drought	Severe heat
p-Hydroxybenzoic	4.25[a]	4.40[b]	4.44[b]	5.01[a]	4.58[b]	3.86[c]	3.53[d]	4.57[b]
Syringic	5.06[a]	6.37[b]	5.97[b]	6.03[ab]	5.68[c]	5.32[cd]	5.04[d]	6.35[a]
Vanillic	7.99[a]	9.55[b]	8.47[cd]	8.82[bc]	8.14[d]	8.57[c]	9.43[a]	9.23[ab]
p-Coumaric	14.49[a]	14.56[b]	13.47[b]	13.5[b]	12.78[c]	12.88[c]	13.13[bc]	21.42[a]
Ferulic	521.91[a]	602.50[b]	540.19[d]	571.47[b]	540.5[d]	544.07[cd]	628.06[a]	550.25[c]
Sinapic	46.71[a]	60.06[b]	60.43[a]	58.63[ab]	58.34[b]	49.32[d]	38.59[e]	55.02[c]
TPAs	600.44[a]	697.44[b]	632.98[d]	663.48[b]	629.96[d]	624[d]	697.78[a]	646.83[c]
